# Reliability Generalization Meta-Analysis of Internet Gaming Disorder Scale

**DOI:** 10.3390/healthcare10101992

**Published:** 2022-10-11

**Authors:** Júlia Gisbert-Pérez, Manuel Martí-Vilar, César Merino-Soto, Miguel Vallejos-Flores

**Affiliations:** 1 Departamento de Psicología Básica, Facultad de Psicología y Logopedia, Universitat de València, 46010 Valencia, Spain; 2 Instituto de Investigación de Psicología, Universidad de San Martín de Porres, 15026 Lima, Peru; 3 Facultad de Psicología, Universidad Nacional Federico Villareal, 15088 Lima, Peru

**Keywords:** IGDS, internet gaming disorder, meta-analysis, reliability

## Abstract

The objective of this study was to carry out a reliability generalization meta-analysis of the Internet Gaming Disorder Scale (IGDS) to find out if it presents adequate values that justify its application in its four versions: original and abbreviated with dichotomous or ordinal response. A systematic review including 26 studies that apply this instrument from 2015 to June 2022 was conducted. For each version, a reliability generalization meta-analysis was performed with the random coefficients (RC) and varying coefficients (VC) model. Results showed reliability levels > 0.80 in the ordinal versions (IGDS9P and IGDS27P) and in the dichotomous 27-item version (IGDS27D), while the dichotomous 9-item version (IGDS9D) produced levels > 0.70. High heterogeneity was found in all versions (I^2^ > 95%; R_b_ > 90%). Cronbach’s α means with both models (RC-VC) did not differ significantly except for the IGDS9D version. It is concluded that, considering the dependence of the results on sample size, abbreviated versions do not guarantee that reliability remains acceptable, and dichotomous versions may provide limited but acceptable reliability. Finally, IGDS27P version is recommended in contexts where high precision is required.

## 1. Introduction

### 1.1. Literature Review

Since the inclusion of Internet gaming disorder (IGD) in the DSM-5 [1], a set of controversies and opportunities arose for mental health professionals regarding the evaluation and intervention of IGD [2,3]. This incorporation reinforced the development and adaptations of psychometric instruments that evaluate the IGD, presenting a set of proposals based on DSM-5 diagnostic criteria [4].

According to DSM-5, IGD is characterized by a pattern of persistent and recurrent gaming behavior that leads to clinically significant impairment or discomfort over 12 months, in which five or more symptoms are present such as preoccupation with gaming, withdrawal symptoms when not gaming (sadness, anxiety, and irritability), tolerance, inability to reduce or stop gaming, giving up other pleasurable activities, continuing to game despite problems, misleading family or others about the amount gaming time, using gaming to relieve negative moods, and risking or losing a job or relationship over gaming [1]. Although this disorder is labeled “online” due to its association with specific online games, it can also develop with offline games [1] (p. 796). It is also included in the ICD-11 as a “video game disorder” [5].

In a systematic review and meta-analysis of IGD prevalence in 17 countries [6], the worldwide prevalence was estimated to be 3.05%. These rates exceed the prevalence of problem gaming, and are similar to those of some substance use disorders and obsessive-compulsive disorder [6].

IGD usually begins in early adolescence [7,8]. During adolescence, there are higher rates of gaming-related problems compared to older age groups [6,9,10,11]. Thus, the pooled prevalence of IGD is 4.6% in adolescent samples [12], with higher rates in men (6.8%) than in women (1.3%). Regarding the geographical context, Asia and North America report a higher prevalence of IGD in adolescents (9.9% and 9.4%, respectively), who also present the highest rates of video game use [13].

However, the prevalence of problematic video game use among adults should be considered. In an intergenerational prevalence study [11], IGD rates were compared among 847 Millennials, Generation X’ers, and Baby Boomers from Hong Kong. The prevalence of IGD increased in the younger generations, being a worrying phenomenon both in Millennials (7.4% of the general sample) and in Generation X (1.3% of the general sample).

With the COVID-19 pandemic, there has been an increase in the number of video game users and related phenomena, such as video game streaming [14]. However, cases of Internet gaming disorders have also increased by between 1–2.5% [15]. This highlights the current importance of the comprehensive and intergenerational study of this phenomenon and the development of reliable diagnostic instruments for IGD.

Since the introduction of IGD in the DSM-5, different instruments have been developed for its diagnosis, such as the Internet Gaming Disorder Test-IGD-20 [16], the Questionnaire of Experiences Related to Video Games (CERV) [17], or the Internet Gaming Disorder Scale (IGDS) [9], among others. This study focuses on the IGDS of Lemmens et al. [9] as it provides greater possibilities in terms of response format (dichotomous and polytomous version), extension (27 or 9 items), and multiple adaptations to other languages [18,19,20].

The IGDS is an instrument based on the nine DSM-5 IGD diagnostic criteria: preoccupation, tolerance, withdrawal, persistence, escape, problems, deception, displacement, and conflict [1]. Since the initial proposal, four variants have been presented: a 27-item scale with a polytomous response or IGDS27P (Likert 0–6), a 27-item scale with dichotomous responses or IGDS27D, a 9-item scale with a polytomous response or IGDS9P (Likert 0–6) and a 9-item scale with a dichotomous response or IGDS9D [9]. The psychometric properties found for all the scales were evaluated in samples of adolescents and young adults, finding high internal consistency in all of them (coefficient α): IGDS27P (>0.94), IGDS27D (>0.93), IGDS9P (>0.95), and IGDS9D (>0.83) [9]. The evidence for validity was acceptable, as well as evidence for content validity [18], internal structure [9,18,19,21], and validity in relation to other variables [9,18,19,22].

Regarding its structure, the unidimensionality of the scale has been reported in some studies [9,18,21] and multidimensional in others [19]. For the diagnosis of gaming disorder, the cut-off point recommended and established by researchers is to present five or more criteria during 12 months, based on the recommendation of the DSM-5 [9]. This criterion, assumed from the DSM-5 (>5), showed high specificity and adequate sensitivity [23]. These authors, assuming this cut-off point, identified three types of players: normal, risk, and disordered [9,21].

However, despite its frequent use, no study reports its average reliability across multiple application studies. The need for a study of these characteristics adds to the fact that some studies report low-reliability values [20,23,24], and therefore, the reliability scores of the instrument may not justify its use. Reliability generalization meta-analyses are used to statistically integrate reliability estimates calculated in different applications of an instrument. In addition, they provide information on how different sample characteristics affect the variation in an instrument’s reliability indices [25]. It should be considered that some aspects can increase the variance error, affecting reliability. Some authors highlight the fatigue in the participants [26], the lack of monitoring in massive applications, or the induction of the validity of the instrument [27,28], among others. Quality controls and specifying the conditions of application contribute to the reliability of the instrument scores [29].

Having a meta-analysis of an instrument that presents good reliability is useful both for a good diagnosis and for providing adequate care to users who require it [30]. After a previous search, no examples of this type of meta-analysis have been found for the IGDS. However, while it was being carried out, a study was detected that included a reliability generalization meta-analysis of the IGDS [31], with which there are substantial differences in the included versions of the IGDS, the meta-analytic models employed, the sample size, and the sample size, and the assessment of heterogeneity. Therefore, this study includes the four main versions of the IGDS, as well as more models and reliability and heterogeneity estimators, and discusses which of the four versions may be more reliable. A more comprehensive differentiation between the two studies is included in Appendix A.

Given that IGD usually begins and develops during adolescence, such a study could benefit clinical practice by providing evidence of the reliability of a diagnostic tool for IGD, as well as promoting the prevention and early treatment of IGD.

### 1.2. Goal Setting

To carry out this study, a main research question was posed: does the IGDS in its four versions present adequate reliability values to justify its application? Consequently, the main objective of this study is to carry out a reliability generalization meta-analysis to estimate the internal consistency of the IGDS in order to know whether it presents adequate values that justify its application in all its versions. Likewise, it is intended to analyze whether the reliability indices are affected by the characteristics of the studies. It was hypothesized that the IGDS, in its four versions, would continue to be an instrument with good internal consistency reliability.

## 2. Materials and Methods

### 2.1. Information Sources

After not identifying a systematic review or meta-analysis similar to the one proposed, a systematic review was carried out following the PRISMA 2020 method [32] in the Web of Science (WoS, Main Collection), PsycInfo, Scopus, and Dialnet databases.

### 2.2. Eligibility Criteria

A protocol was registered in PROSPERO, with the identification code CRD42022330840. For screening, the following inclusion criteria were proposed: (a) original empirical studies that apply the IGDS, (b) include the IGDS in the original or translated language, (c) include the IGDS in the original or reduced version, (d) validations and adaptations of the IGDS, and (e) report the reliability of the instrument using Cronbach’s α or another indicator. It was not considered to limit the search to the type of sample, since the instrument has been adapted to different populations. Likewise, those studies that met any of the following exclusion criteria were discarded: (a) not using the IGDS and (b) book chapters or books.

### 2.3. Search Strategy

First, manuscripts using IGDS instrument [9] were identified in three iterations. The search was carried out in July 2021 and was updated in June 2022. This process was carried out by one of the authors and corroborated by another through the Covidence tool.

#### 2.3.1. First Iteration

The first search was performed on the Web of Science (WoS Main Collection), PsycInfo, Scopus, and Dialnet databases. On all bases, the term “Internet Gaming Disorder Scale” was introduced to include all those works that contained said instrument. Given the generality of the search profile, the results were refined by including only the works between 2015 and June 2022, both inclusive, and written in English and Spanish.

The search was limited to the field of psychology. In the WoS database, the search was limited to the categories “social sciences”, “psychology”, “applied psychology”, “clinical psychology”, “developmental psychology”, “experimental psychology”, “multidisciplinary psychology”, and “psychology Social”. In Scopus, the fields of study “psychology” and “social sciences” were selected. In Dialnet, it was not limited by the field of study. At PsycInfo, the field of study was also not limited by being a psychology database.

#### 2.3.2. Second Iteration

The second search was manual, reviewing the references of the studies found in the first search.

#### 2.3.3. Third Iteration

A second manual search was performed in Google Scholar to expand the sample.

### 2.4. Selection of Studies

Once duplicates were eliminated with RefWorks bibliographic manager, the Covidence Software was used to carry out a blind peer review by title and abstract, and full text, following the eligibility criteria. In longitudinal studies or those that included more than one measurement performed on the same subjects, the first study was selected, or in its exception, the first that reported a reliability coefficient. In contrast, studies using more than one sample and their reliability values were considered independent samples.

### 2.5. Data Extraction

The reliability coefficient of the selected studies (α, McDonald’s omega (ω), or test-retest) was manually extracted. Both reported (i.e., the study’s coefficient) and induced reliability values were considered. In this scale, the use of the single score or total α (although there are several specific dimensions) is indicated and was used for the correlations of the original study. Therefore, no study reports the reliability of the 9 dimensions of the scale.

Regarding the induced reliability, it was subdivided into three categories: omitted, vague, and precise [33]. It was considered omitted when no reliability coefficient was reported in the study, vague when “good” reliability was expressed by citing other previous studies, and precise when the exact value of a previous study was reported.

From the studies with reported reliability values, information was extracted regarding the year of publication, version of the IGDS, the language of the IGDS, country of application, application method, sample size, general characteristics of the sample, and classification of the gamers in the sample (include only players or players and non-players), mean age, mean IGDS scores and standard deviation, percentage of women in the sample, mean weekly gaming hours, data collection method, adjustment indices, use of test-retest, presence of statistical validation of the IGDS, and percentage IGD+ in the sample. Data extraction was performed with Covidence and Microsoft Excel.

### 2.6. Analysis

After obtaining the sample, a reliability generalization meta-analysis was performed for each version of the IGDS following the recommendations of the REGEMA checklist (Appendix B).

#### 2.6.1. Description and Evaluation of α Coefficients

The adequacy of the reliability in each study, and the mean α coefficient, were evaluated by comparing their confidence intervals (95%) obtained against a null value [34]. This null value was established in two values of the coefficient α: 0.70 and 0.80. These values were chosen because they are usually minimum criteria to generically determine the appropriateness of a reliability coefficient [35].

#### 2.6.2. Modeling

Due to the characteristics of the study that could influence the conclusions (i.e., inter-study and intra-study heterogeneity, and the size of the selected studies), the modeling decision was oriented towards two approaches: random coefficients (RC model) [36] and variant coefficients (VC model) [37].

Random coefficients model. There are several contextual factors (e.g., a large number of measures applied to an examinee, evaluation monitored by an examiner or without monitoring, etc.), individual variables (e.g., mood, motivation, effort, personality attributes, etc.), data quality (e.g., multivariate outliers, response trends, etc.), and the interaction between them, which can produce variability in the estimation of the reliability of the obtained score [38,39]. Thus, the first model for meta-analyzing the α coefficients of the IGDS was random effects (RC) [36]. RC has several underlying assumptions: first, the estimates obtained vary from study to study, due to actual differences between studies, and due to sampling variation. Second, the study sample came from a random selection from a normally distributed population of α coefficients, which implies that this is an overpopulation. Third, to generalize the results to future studies not similar to this study, the RC model is generally accepted as the recommended option, and is one of the preferred goals of research [40].

For the estimation of the amount of variance between studies (τ^2^) [41], the restricted maximum likelihood (REML) method [42] was implemented, with the Knapp–Hartung modification [43] was applied to the statistical test of variability (Q) [44], τ^2^, and confidence intervals. This method is based on the Student’s t-distribution and tends to provide more robust estimates under various data conditions [45]. The measures of heterogeneity of the selected studies were: (a) the statistical test *Q* [44], with the significance level set at 0.10 [46], and (b) estimators of the size of heterogeneity: I^2^ [47], R_b_ [48], and CV_b_ and CV_w_ [48] as measures of between-study and intra-study variability, respectively [48].

Due to the potential bias produced in the estimation of I^2^ in meta-analyses with a small number of studies [49], its interpretation mainly used its confidence intervals. Two qualitative criteria used to interpret I^2^ were: The first, by Deeks et al. [50] and Higgins et al. [47]: <I^2^ = 40% (“could be small”), I^2^ = 30–60% (“could be moderate”), I^2^ = 50–90% (“could be moderate”), and I^2^ = 75–100% (“could be substantial”). The second, by Higgins et al. [51]: I^2^ < 25% (trivial), I^2^ ≥ 25% (minor), I^2^ ≥ 50% (moderate), and I^2^ ≥ 75% (substantial).

According to the recommendation of Sánchez-Meca et al. [52] and Romano et al. [53], under the RC model, the α coefficients were transformed with the Bonett method [54], $L_{i}=Ln\left(1-\alpha \right)$, and their sampling variance [54] was obtained with: $V_{i}=\frac{2J}{\left(J-1\right)\left(n_{i}-2\right)}$, in which J: number of items and n_i_: sample size of the study. On the other hand, the weighting of the studies to obtain the αmean was conducted with the general approach of creating weights based on the inverse of the variance [55]. To assess the independence between the size (i.e., number of participants) and the α coefficient of the studies [37,56], both parameters were correlated. Respectively, for IGDS9D, IGDS9P, IGDS27D, and IGDS27P, the following was obtained: r = 0.149 (*p* = 0.53), r = −0.213 (*p* = 0.78), r = 0.382 (*p* = 0.61), and r = −0.793 (*p* = 0.108). According to the lack of statistical significance, the weights could be applied with little apparent risk of bias in the estimation of the αmean in the IGDS9D, IGDS9P, and IGDS27D, but the interpretation of the results requires caution in IGDS27P due to the size of the correlation.

Varying coefficients model. Although meta-analytic research usually uses the RC model, the analysis was also conducted with the varying coefficients (VC) model [37]. This model was chosen due to: (a) the unlikely fulfillment of the assumption of normality of the hypothetical population of α coefficients, (b) the actual absence of random selection of manuscripts, and (c) the small number of selected studies (i.e., less than 6 in IGDS9P, IGDS27D, and IGDS27P). These are conditions that make it difficult to justify the RC model, particularly when the identification of a well-defined population of studies is problematic [37]. Specifically, in our study, variability is observed in multiple factors of the sample. Firstly, there are differences in gamer conceptualization. On the one hand, some studies define a gamer according to a minimum game frequency (e.g., playing at least once a month [9,21,57,58,59,60,61]), while others only consider as gamers those who currently play [18,19,20,24,62,63,64,65,66,67]. On the other hand, some studies did not specify what they considered as a gamer, since in most of these cases the diagnosis of IGD was not their main objective [15,20,68,69,70,71,72,73,74]. Differences are also observed in terms of the characteristics of the samples, sometimes including players and non-players or exclusively gamers. In some studies, game intensity (hours and days of game), gamer profiles (players for fun, amateur, professionals, etc.), and video game genre (e.g., MMORPG) were considered. Methodological variability was also observed. Finally, both the size and age groups of the study samples are variable, ranging from 204 to 2078 participants, including adolescents, young adults, adults, and the general community. Consequently, heterogeneity is present in different combinations of the aforementioned variables.

VC model is an appropriate approach when the number of meta-analyzed studies is small (<30) [52], when strong heterogeneity is present (in the Results section, this is observed), and when there was no randomized extraction of studies [75]. In contrast to the RC model, the generalizability of the VC results is oriented to a population of studies similar to those that were selected [52]. VC does not assume compliance with the common assumptions of fixed effects and random coefficient methods [76]. In the VC method, the log-transformation is applied to αmean (ln [1 − α_mean_]) [37] to stabilize the variance [54], and the studies are not weighted to obtain the mean meta-analytics.

#### 2.6.3. Sources of Heterogeneity

Due to the small number of studies analyzed (<25), the identification of sources of heterogeneity in the IGDS with the largest number of meta-analyzed studies (i.e., IGDS9D, *n* studies = 20) was explored with K-means cluster analysis, within a dependency *cluster–covariate analysis* [77]. Accordingly, (a) strictly exploratory clusters were identified with the K-means procedure, and (b) these clusters were compared with the existing natural clusters in the studies. This comparison was made using the χ^2^ independence test, and the Cramer-V effect size estimator. The similarity found in this comparison would suggest the substantive interpretation of these new groupings and avoid random capitalizing. The descriptive variables were: the language of the scale (original and English dichotomous classification), mode of application, age characteristics of the sample (adolescents, young people, adults, or general community), and the condition of gamers in the sample (only gamers or mixed samples with gamers and non-gamers) (Appendix C). Differences in mean alpha estimates for each group of studies were estimated using Bonnet’s method [37], based on the confidence interval of the differences (CI ∆_diff_).

In the versions with smaller number of meta-analyzed studies (i.e., IGDS9P, IGDS27D, and IGDS27P; in all *n* ≤ 5), subgroup identification was performed on a quantitative–qualitative basis, and within *qualitative evidence synthesis* (QES) framework [78]. This was conducted to identify the distinguishing characteristic of the studies that could be associated with the variability of the αmean coefficient. The procedure followed was: (a) quantitative identification of homogeneous groups with K-means cluster analysis, (b) content exploration of the identified groups by K-means analysis (i.e., qualitative examination of the characteristics of their studies from their descriptive variables: instrument language, etc.), (c) assignment of apparent quality that distinguishes these identified groups, and (d) reproducibility evaluation of the three previous steps (independently by one of the authors).

#### 2.6.4. Outliers and Robust Estimation

As part of the heterogeneity assessment, outliers were detected for each study, and the mean α was robustly reestimated excluding them. For each study, its impact on τ^2^ was also estimated, using the V_RATIO_ and TAU_RATIO_ statistics [79]; the cut-off point Q (Q_vratio_ and Q_tauratio_) to identify the strength of the impact of each study (V_RATIO_ > Q_vratio_; TAU_RATIO_ > Q_tauratio_) was established with 1000 bootstrap samples [79].

Regarding the software used, the following R programs were used: RC modeling with *metafor* [80], VC modeling with *vcmeta* [81], the impact assessment of each study on variability was conducted with *boutliers* [79], outlier detection and robust estimation were conducted with the *dmetar* [82], alternative measures of heterogeneity (R_b_, CV_b_, CV_w_) were obtained with the R *hetmeta* [48], and K-means cluster analysis with R *stats* [83].

## 3. Results

### 3.1. Results of the Study Selection Process

**Figure 1:**
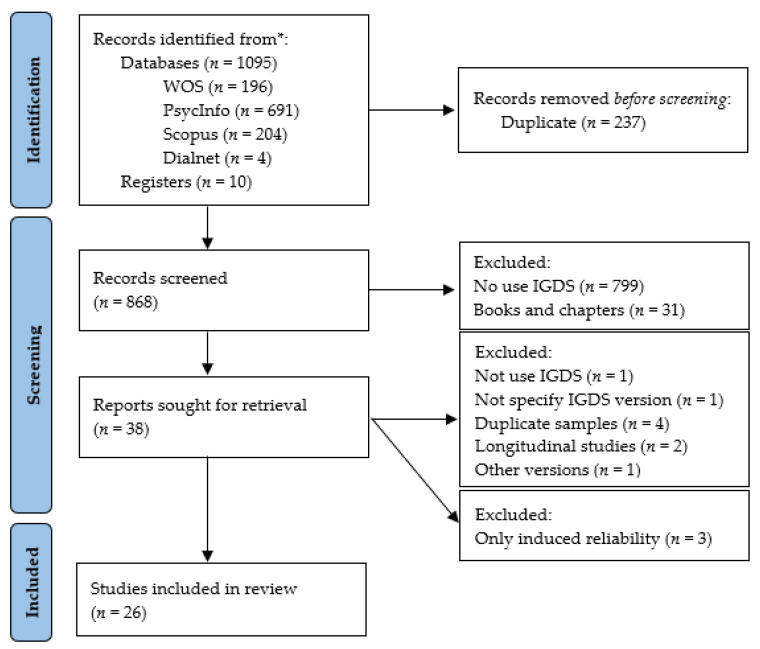
Flowchart of the selection and screening process of the systematic review articles according to the PRISMA 2020 statement.

The identification, screening, and selection process carried out according to PRISMA 2020 [32] is detailed in Figure 1.

First, using the database tools, a total of 1095 articles were identified, including 204 from Scopus, 196 from WoS, 691 from PsycInfo, and 4 from Dialnet. Additionally, 10 articles were identified in a second (*n* = 3) and third (*n* = 7) iteration. After removing duplicates (*n* = 237), the remaining 868 articles were screened for eligibility criteria. In total, 38 articles were selected to read the full text. Nine articles were excluded: for not administering the instrument (*n* = 1), for not specifying IGDS version (*n* = 1), for administering a version whose answers are provided by third parties (*n* = 1), second measures from longitudinal studies (*n* = 2), and by using repeated samples (*n* = 4). Of the 29 studies, 3 studies only include induced reliability values (omitted (*n* = 2) and precise (*n* = 1)). Only articles that reported reliability (*n* = 26) were included for the meta-analysis. These studies were divided according to the version of the IGDS administered. As mentioned, the studies that presented several independent samples that reported the reliability values and the N of each one of them were coded as independent samples. The distribution was as follows: IGDS27P (*n* = 5), IGDS27D (*n* = 4), IGDS9P (*n* = 4), and IGDS9D (*n* = 20) (Appendix C).

### 3.2. Description and Evaluation of the Level

Results are presented in Table 1. Regarding IGDS9D, 60% (*n* = 12) of the studies produced an alpha coefficient > 0.70, while the rest were below this level (20%, *n* = 4) or had an inconclusive result (20%, *n* = 4), because the confidence interval of the difference includes zero. Meanwhile, less than half of the studies produced coefficients > 0.80. Regarding IGDS9P and IGDS27P, all random-effects coefficients > 0.80. Finally, IGDS27D was predominantly > 0.80.

### 3.3. Random-Effects Model

#### 3.3.1. Mean Reliability

The size distribution of each study ranged from 204 to 3938 (M = 941.1, Md = 780). Based on 20 studies (n_total_ = 20) for IGDS9D, the mean reliability was higher than 0.70. In contrast, the mean point estimates for the remaining versions IGDS9P (n_total_ = 4), IGDS27D (n_total_ = 4), and IGDS27P (n_total_ = 5) were found to be higher than 0.90. In the population variability range (95% CI), the coefficients varied between approximately 0.75 and 0.97, higher than 0.70. The prediction intervals for IGDS9D, IGDS9P, and IGDS27D, placed the mean reliability at low levels, except for the mean reliability of IGDS27P, in which the predictivity of the mean coefficient is above 0.90.

#### 3.3.2. Robust Estimate

Outliers were only detected in the IGDS9D studies, specifically nine studies [9,15,20,23,24,59,64,65,69]. With the remaining 11 studies [18,20,61,62,63,68,71,73,74], the αmean was 0.781 (se = 0.05), 95% CI = 0.758, 0.802 (95% PI: 695, 843). The heterogeneity of this robust estimate was statistically significant (Q = 101.49, df = 11, *p* < 0.001), and, also, had a high I^2^ = 89.9% (95% CI = 78.8%, 96.8%), even with high intra-studies (CV_W_ = 0.603) compared with between-study variability (CV_b_ = 0.106, 95% CI = 0.106, 0.108).

### 3.4. Varying Coefficients Model

#### 3.4.1. Heterogeneity Estimation

The smallest part of the heterogeneity came from random variation (<0.10%), while the strong degree of between-study heterogeneity (I^2^ > 95%; R_b_ > 90%) was predominant in the analysis of all IGDS versions. Between-study variability (CV_b_) was trivial in the IGDS27P studies, and was similar in the rest of the IGDS versions (CV_b_ between 0.189 and 0.206), while the variability from intra-study differences was strong in the IGDS9D, compared to the rest (CV_W_ between 0.383 and 0.473) (Table 2).

#### 3.4.2. Exploratory Analysis

IGDS9D. In the cluster–covariate analysis [77], two exploratory solutions were identified in the IGDS9D (Table 3), one of three groups (three Clusters: group 1 = 6 studies, group 2 = 2 studies, group 3 = 12 studies), and another of two groups (two clusters: group 1 = 12 studies, group 2 = 8 studies). The Χ^2^ independence test did not reject the null independence hypothesis between these two solutions and the existing groupings in the data (language of the scale, language, mode of application, sample, and gamer condition; see Table 4). These results suggest that the association found (Cramer-V) may be included in the sampling variation. Advancing in a qualitative evidence synthesis (QES) framework [78], regarding the solution of three groups: in cluster 1, 2 articles share samples of young people (under 20 years of age) in which only gamers are included, in cluster 2, all agree on the administration of the IGDS in European languages and online administration, and in cluster 3, no similarities are found. Between clusters, there are no clear differences in the variables studied.

IGDS9P. The minimum interpretable solution was two groups (see Table 3). Qualitative analysis of the similarity between this clustering and the study characteristics suggested that cluster 1 were similar in including general population, with similar mean ages, and gamer-only samples (although the report of the Lei et al. [19] study is missing). The difference in α coefficient between the cluster 1 studies (study 1 and 2; α mean = 0.94), with the cluster 2 (study 9 and 18; α mean= 0.87) can be established as statistically significant, but also as small (∆α = 0.07; 95% CI ∆_diff_ = 0.05, 0.08), because the range of the difference (95% CI ∆_diff_) was small and close to 0.0.

IGDS27D. The minimum interpretable solution was two unbalanced groups (see Table 3). Qualitative analysis of the similarity between this grouping and the characteristics of the studies suggested that the studies of cluster 1 only include gamers in their samples, compared to the study of cluster 2, which has a mixed sample. However, the difference in α coefficient between the study identified as cluster 2 (study 4, α = 0.81) and the rest of the three studies (αmean = 0.92), can be established as trivial, (∆α = −0.11, 95% CI _∆diff_ = −0.13, −0.08).

IGDS27P. The minimum interpretable solution was two unbalanced groups (see Table 3). Qualitative analysis suggested that the only difference between both groups was the presence of gamers and non-gamers in the sample of the second study. However, the difference in the α coefficient between the study identified as the only member of its group (study 2, α = 0.97), with the rest of the three studies (αmean = 0.95) can be established as trivial, (∆α = −0.01, 95% CI _∆diff_ = −0.01, −0.00).

## 4. Discussion

The objective of this study is to perform a reliability generalization meta-analysis of the IGDS. For this purpose, the internal consistency values of the IGDS in different samples are analyzed, and possible causes of the observed variations are examined [84].

The appropriateness of the alpha coefficients of the retrieved studies depended on instrument length (number of items) and response scaling (i.e., dichotomous and polytomous); both structural characteristics had a clear influence, as all versions of the IGDS using ordinal scaling (IGDS9P and IGDS27P) or the long version with 27 dichotomous items (IGDS27D), almost entirely produced scores with reliabilities >0.80. In contrast, the short version with dichotomous items (IGDS9D) produced levels <0.70.

Two major issues to be elaborated in this discussion can be drawn. Ordinal scales and the larger number of items can be taken into account when choosing the IGDS version. In terms of brevity, the IGDS9P may be the recommended version. In a first look at the influence that each study had on the overall estimation of the degree of heterogeneity of the studies (with V_RATIO_ and TAU_RATIO_) [79], almost all individual studies potentially produced non-negligible changes in the α-mean heterogeneity of each version (Table 1). In the versions with few meta-analyzed studies (IGDS9P, IGDS27D, and IGDS27P), there was an apparent hypersensitivity and consequent Type I error. The studies detected as a source of statistically important impact subsequently showed trivial differences with the rest of the coefficients compared.

Regarding the α coefficient meta-analysis, the point estimates of the mean α produced by the VC and RC model were not substantially different, except for the IGDS9D version (αmean = 0.775; 95% CI = 0.74, 0.80). The rest of versions can achieve a level of accuracy of the scores that is usually considered optimal for group description purposes and basic research [35,85].

In both RC and VC models, IGDS27P was shown to be exceptionally high (lower CI limit > 0.93), while the most widely used version, IGDS9D, remained at an acceptable level (lower CI limit > 0.70), but not optimal compared to the rest of the IGDS versions. One implication of these specific results is that the IGDS27P may be the best option for using highly reliable scores, particularly when the context of IGDS use demands this level of precision, such as the classification of individuals, and the differentiation of individual gamer vs. non-gamer, etc. On the contrary, with both estimated models (i.e., CR and VC), IGDS9D showed acceptable levels of reliability, but they are likely to be unacceptable when high precision in the interpretation of its scores is required, as in clinical practice [25]. Due to the number of meta-analyzed studies (n_studies_ = 20) in the IGDS9D version, this conclusion is relatively reliable and can be taken as a reference.

The prediction intervals (PI) did not set optimal minimum values in future studies using the IGDS9D, IGDS9P, and IGDS27D. Using the lower limit of the PI as a reference, the lowest estimates obtained (IGDS9D = 0.56, IGDS9P = 0.51, and IGDS27D = 0.48) showed that the scores may contain a high proportion of error variance, even at levels where no instrument would be eligible for research and applied use (<0.60). On the other hand, the upper limit of the PI indicated that the predicted levels can exceed the value 0.85. The best consistently achieved level on this was the IGDS27P, where the error variance was consistently very low. Three implications of these results are that: first, to maintain high and optimal true variance in IGDS scores, the IGDS27P is the best option in the context of high intra-study variability; second, the abbreviated versions of the IGDS (IGDS9D and IGDS9P) do not guarantee that the reliability remains within a range of acceptable use; and third, the dichotomous versions of the IGDS may yield a limited minimum acceptable reliability. Therefore, it is apparent that IGDS27P is the recommended option in a wide range of applications, especially when high precision is required. As a further note here, the confidence intervals generated by RC tended to be wider compared to the Cis generated by VC, a problem intrinsic to the RC model when the number of studies is small [37].

In the robust analysis, the re-estimation of αmean could only be conducted in the IGDS9D, because in the rest of the versions no outliers were detected. The robust αmean obtained was slightly higher (αmean = 781) than the non-robust estimate (αmean = 0.775), as well as the 95% CI was very similar, and therefore both parameters can be considered equivalent. This equivalence may be because the outliers were symmetrically distributed, producing little bias in the estimation [86]. In contrast, the PI was different in the robust analysis (95% PI: 0.695, 843), because the predicted level of the α coefficient indicates an acceptable level of reliability. An implication of this is that the user must consider that the internal consistency of the IGDS9D can be maintained at the level declared in previous paragraphs.

It should be mentioned that the detection of outliers by the method used (i.e., Harrer, et al. [82]) did not have an apparent sensitivity in the studies analyzed for IGDS9P, IGDS27D, and IGDS27P, as no outlier studies were detected. This suggested that the heterogeneity detected could not be explained by the presence of studies with extreme alpha coefficients (i.e., outlier studies). Given the strong heterogeneity found, it is likely that the reason for this lack of sensitivity of the method used [82] was the effect of the small number of these studies in each version (n_studies_ ≤ 5).

Usual (I^2^) and new (R_b_) indicators of heterogeneity converged in pointing out that the amount of variability was strong (>90%), and that an important source seems to come from the intra-study variability (CV_w_) compared with the inter-study variability (CV_b_). The exploration of the heterogeneity between the studies gave an unclear clue as to the sources of the variability of the alpha coefficients. Qualitative inspection suggested that when the size of meta-analyzed studies is small [78] only some studies were an apparent source of differentiation (i.e., the study by Evren et al. [19] on IGDS9P, Zemestani et al. [67] in IGDS27D, and Evren et al. [19] in IGDS27P; Table 3), but this was trivial because the differences produced in their αmean coefficients without these studies were of trivial size.

The exploration of the heterogeneity carried out suggests that there are other possible and realistic explanations for the degree of heterogeneity found. These can be attributed to the characteristics of the individual studies, which may be causally linked to the distance of each alpha coefficient from its αmean. Specifically, methodological or artificial heterogeneity [87] may have played an important and not ignorant role in this heterogeneity. The methodological aspects are specifically focused on the quality of the data, and the calibration of the IGDS in each sample. In the first, possible response biases were not explicitly incorporated into the analytical procedures of the selected studies, which requires a set of a priori decisions about their detection or treatment. These have been termed random responses [88], item content-independent responses [89], insufficient effort response [90], or careless response [91].

There is empirical literature that has shown its effects on a long chain of statistical estimates, such as the spurious variability of responses [38,39], the internal structure of the instrument [39,92], and in general, the prevalence of error Type I and Type II [93]. In the study of behavioral addictions mediated by the web platform, this issue is no less critical compared to other areas of research. There is an explicit call to address it as well [94], more so when a small prevalence of C/IE responses can produce non-ignorable changes in quantitative estimates [95], which are unrealistic for the measurement of the construct of interest [92].

Second, in the selected substantive studies, the internal structure of the IGDS was not explicitly verified, resorting to supporting the validity argument using previous results. This is a problem of inducing the validity of the instrument (specifically, its structure or the interpretation of its scores) from background evidence, but without corroborating it with the available data [27,28,96]. Due to the natural variability of samples and application conditions, it does not seem reasonable to expect that the internal structure of an instrument will remain intact, even more so if the instrument contains several items that capitalize on sampling variability and methodological variability. These changes in the instrument may be expressed in different magnitudes of intra-study or between-study factor loadings on the set of items, the presence of correlated residuals between items, or the emergence of a general method factor. This means that even to obtain a valid measure of the reliability of the scores, this corroboration is required [35,97], and as a general rule, it should be resolved even in non-psychometric studies. As part of good reporting practices, it is proposed to check the internal structure of the IGDS and to report the psychometric adjustment obtained.

Modern reliability estimates aim to calculate other coefficients that tend to better represent the structure of the items of a measure, such as the coefficient ω [98] for congeneric one-dimensional measures [99], that is, with variability in factor loads of the items. This reliability measure was hardly calculated in the selected studies with the IGDS, and it is reasonable to conclude that the reliability estimates obtained with the alpha coefficient are the lower bond of the reliability of the scores obtained by omega [98,99]. A practical implication is that the mean reliabilities of the IGDS versions may be higher than those obtained in the present study. How high? It is not possible to give an approximate or precisely answer, due to the high prevalence of induced validity and the consequent lack of knowledge of factor loads. In practice, it may be advisable to report both coefficients, α and ω.

Finally, in comparison with another meta-analysis analyzing the reliability of various instruments [31], the two polytomous versions of the IGDS (IGDS27P and IGDS9P) show higher reliability scores than those found on other similar instruments. This highlights that these versions may be more suitable for the assessment of the IGD.

### 4.1. Limitations

Firstly, the present study considered that the minimum reasonable evidence to quantitatively addressed the generalization of reliability was the number of studies included (20 in the IGDS9D; between 4 and 5 in the IGDS9P, IGDS27D, and IGDS27P). Although each contributed a substantial number of participants (n_participants_ > 2000), the accumulation of more studies may be required to reach more reliable conclusions about the αmean and sources of heterogeneity. Secondly, the interpretation of the PI can be reliable in the IGDS9D, but with caution in the conclusions of the IGDS9P, IGDS27D, and IGDS27P, due to the small size of the studies included in these three versions. Thirdly, the conclusions about the αmean in each version analyzed must be contextualized by the degree of heterogeneity found, especially in the versions where the number of studies was less than six (i.e., IGDS9P, IGDS27D, and IGDS27P). Adding to this general situation, in our analyzes the number of retrieved studies was small (particularly for the IGDS9P, IGDS27D, and IGDS27P versions), and few studies cannot serve to reliably identify sources of heterogeneity [100]; for this reason, heterogeneity was conducted in an exploratory manner, combining quantitative and qualitative means to maximize the opportunity to recognize these sources. Fourthly, it is possible that, within a general framework of sensitivity, it is necessary to implement new estimators [101], but it is adapted to the study of reliability generalization, or to take advantage of the Bayesian approach [102] in the area of the generalization of the reliability of the IGDS.

### 4.2. Practical Implications and Future Research

The continuous evolution of video game consumption, the increase in rates of problematic consumption, and the expansion of consumption beyond adolescence highlight the social relevance of the IGD study. Carrying out a reliability generalization meta-analysis of evaluation or diagnostic instruments guides professionals on which scales are more reliable to evaluate a certain construct, as well as in what circumstances [25]. Given that psychological intervention in social settings covers all social sectors (children, adolescents, young adults, etc.), the availability of reliable instruments can be helpful in prevention, diagnosis, evaluation, and psychological intervention, as well as in choosing therapeutic strategies. Therefore, due to the onset and prevalence of IGD in adolescence, the results of this study have implications for clinical practice, confirming the accuracy of the instrument for the early detection and intervention of this disorder. All of this has a direct positive impact on the promotion of well-being in this vital sector and beyond, as well as the promotion of prevention at earlier ages.

On a practical level, after the results are obtained, the IGDS27P is shown to be the most recommendable version for contexts where high reliability is required. However, despite the more frequent use of the IGDS9D, the use of its polytomous version is also recommended when the use of an abbreviated version is required.

For future research, it is proposed to continue with the study of the IGD in different countries with differential video game consumption or to analyze variables that could positively or negatively influence the development of IGD. Moreover, after the sample size is achieved, it would be important to re-perform a meta-analysis to generalize the reliability of the same instrument or even include more instruments measuring this construct, in order to corroborate the results found, as well as to study the psychometric properties of these instruments.

## 5. Conclusions

The present work carried out a reliability generalization meta-analysis of the IGDS from 2015 to June 2022, considering its four versions, original and abbreviated with dichotomous and polytomous responses. In the meta-analysis, it was observed that a large part of the sample of studies reviewed lacked some data and presented high heterogeneity, which made it difficult to correctly interpret the generalization of its reliability. The results of the study suggested that to achieve high-reliability scores it was advisable to use the IGDS with ordinal response, for both the 9-item and 27-item versions, the latter being the most appropriate when high precision is required. In contrast, the IGDS9D version had the lowest reliability and could compromise the interpretation of its scores, and was, therefore, the least recommended. These conclusions are dependent, however, on the size of the meta-analyzed studies in each version, and considering that the version with nine dichotomous items was the one with the largest meta-analyzed studies, this conclusion may be more generalizable. Regarding the exploration of heterogeneity, high variability was found, and it was not possible to classify the studies based on the characteristics of the registered data (language of the scale, mode of application, age characteristics of the sample, and status of gamers in the sample). For this reason, a qualitative analysis was carried out that highlighted as a possible cause of variability the use of samples of only gamers in the original and abbreviated polytomous and original dichotomous versions, but this apparent source of differentiation was trivial. Regarding other sources of heterogeneity, the studies did not include information to identify methodological variability (e.g., response bias control, outliers, etc.), which could play an important role in the heterogeneity found. Finally, the need to strengthen adequate reliability reporting practices in primary studies to optimize their reporting is highlighted.

## Figures and Tables

**Table 1:** Characteristics of the reliability levels of the articles included in the meta-analysis.

	N	*α*	se	95% CI α		Levels		Impact on Variability			
				Ll	UL	>0.70	>0.80	V_RATIO_	Q_vratio_	TAU_RATIO_	Q_tauratio_
**IGDS9D**	18,828										
Study 1	989	0.830	0.00	0.814	0.845	Y	Y	10.07 *	0.87	10.02 *	0.82
Study 2	394	0.850	0.00	0.826	0.871	Y	Y	10.04 *	0.88	0.99 *	0.83
Study 3	1020	0.820	0.01	0.803	0.836	Y	Y	10.08 *	0.89	10.03 *	0.84
Study 4	204	0.610	0.00	0.523	0.684	N	N	0.92	0.92	0.88	0.88
Study 5	708	0.810	0.04	0.788	0.830	Y	NC	1.09 *	0.88	1.04 *	0.83
Study 6	354	0.740	0.01	0.697	0.778	NC	N	1.10 *	0.88	1.05 *	0.83
Study 7	538	0.730	0.02	0.694	0.763	NC	N	1.09 *	0.89	1.04 *	0.84
Study 8	257	0.820	0.01	0.784	0.851	Y	NC	1.08 *	0.88	1.03 *	0.83
Study 9	310	0.840	0.01	0.811	0.865	Y	Y	1.06 *	0.89	1.00 *	0.84
Study 10	855	0.810	0.01	0.790	0.828	Y	NC	1.09 *	0.89	1.04 *	0.83
Study 11	1306	0.690	0.01	0.664	0.714	NC	N	1.04 *	0.89	0.99 *	0.84
Study 12	351	0.810	0.01	0.778	0.838	Y	NC	1.09 *	0.90	1.04 *	0.84
Study 13	1001	0.660	0.01	0.627	0.691	N	N	0.98 *	0.90	0.93 *	0.84
Study 14	1081	0.790	0.01	0.771	0.808	Y	NC	1.11 *	0.86	1.05 *	0.81
Study 15	2078	0.740	0.01	0.723	0.756	N	N	1.10 *	0.88	1.05 *	0.83
Study 16	1121	0.830	0.00	0.815	0.844	Y	Y	1.07 *	0.86	1.02 *	0.81
Study 17	3938	0.840	0.00	0.832	0.847	Y	Y	1.06 *	0.88	1.00 *	0.82
Study 18	762	0.780	0.00	0.756	0.803	Y	N	1.11 *	0.87	1.06 *	0.81
Study 19	777	0.720	0.01	0.689	0.748	NC	N	1.09 *	0.88	1.03 *	0.83
Study 20	784	0.560	0.01	0.512	0.604	N	N	0.68	0.90	0.63	0.85
**IGDS9P**	2787										
Study 1	923	0.95	0.002	0.945	0.955	Y	Y	1.09 *	0.26	0.81	0.26
Study 2	457	0.93	0.005	0.920	0.939	Y	Y	1.73 *	0.21	0.45	0.21
Study 9	1029	0.85	0.007	0.836	0.863 *	Y	Y	0.61 *	0.25	1.30	0.25
Study 18	378	0.89	0.009	0.872	0.906	Y	Y	1.87 *	0.21	1.42	0.21
**IGDS27D**	2919										
Study 1	989	0.93	0.003	0.9234	0.936	Y	Y	1.64 **	0.27	1.23 *	0.17
Study 14	1026	0.90	0.004	0.8908	0.908	Y	Y	2.02 *	0.17	1.52 *	0.11
Study 16	423	0.93	0.004	0.9198	0.939	Y	Y	1.64 *	0.18	1.23 *	0.10
Study 26	481	0.81	0.012	0.7842	0.833 *	Y	NC	0.12	0.22	0.09	0.15
**IGDS27P**	2454										
Study 1	923	0.95	0.001	0.95	0.96	Y	Y	0.31	0.64	0.20	0.49
Study 2	457	0.94	0.002	0.93	0.94	Y	Y	1.03 *	0.64	0.80 *	0.49
Study 5	315	0.97	0.002	0.96	0.97	Y	Y	1.63 *	0.78	1.33 *	0.60
Study 13	272	0.96	0.003	0.95	0.96	Y	Y	1.62 *	0.78	1.32 *	0.60
Study 19	487	0.96	0.003	0.95	0.96	Y	Y	1.64 *	0.64	1.33 *	0.49

α: Cronbach’s α reliability; n: sample size; se: standard error; >.70, >.80: Levels for qualify α (Y = yes; N = no; NC = non conclusive); V_RATIO_ y TAU_RATIO_: impact indexes of each study, on the variability of *α*_mean_; Q_vratio_, Q_tauratio_: cutoff points for V_RATIO_ y TAU_RATIO_ (1000 bootstrap samples). * *p* < 0.05. ** *p* < 0.01.

**Table 2:** Meta-analytical estimates.

	K	α_+_	95% CI		Heterogeneity					
			Confidence LL, UL	Prediction LL, UL	Q (df)	τ (τ^2^)	I^2^ (CI 95%)	R_b_ (CI 95%)	CV_b_ (CI 95%)	CV_w_
**Random coefficients model**										
IGDS9D	20	0.775	0.74, 0.80	0.56, 0.88	726.40 ** (11)	0.307 (0.094)	97.43 (95.5, 98.8)	96.1 (96.2, 96.4)	0.206 (0.20, 0.20)	0.864
IGDS9P	4	0.912	0.81, 0.95	0.51, 0.98	279.53 ** (3)	0.488 (0.233)	98.55 (95.4, 99.8)	98.4 (98.3, 98.4)	0.198 (0.19, 0.20)	0.408
IGDS27D	4	0.908	0.79, 0.95	0.48, 0.98	174.98 ** (3)	0.465 (0.217)	98.63 (95.7, 99.9)	98.5 (98.3, 98.6)	0.201 (0.19, 0.20)	0.383
IGDS27P	5	0.958	0.943, 0.969	0.913, 0.980	79.41 ** (4)	0.244 (0.05)	92.99 (80.6, 99.1)	92.2 (93.1, 93.5)	0.07 (0.07, 0.07)	0.473
**Varying coefficients model**										
	**K**	**α** **_’+_**	**Confidence** **LL, UL**							
IGDS9D	20	0.764	0.755, 0.775	-	-	-	-	-	-	-
IGDS9P	4	0.905	0.889, 0.991	-	-	-	-	-	-	-
IGDS27D	4	0.892	0.885, 0.899							
IGDS27P	5	0.958	0.955, 0.960							

k: number of studies; GF: k–1 degrees of freedom; LL, UL: lower and upper limits; α_+_: mean coefficient α; τ^2^: between-studies variance estimated using restricted maximum likelihood. ** *p* < 0.01.

**Table 3:** Exploratory analysis results.

IGDS9D	Kmeans 1 (n_g_ = 3)	Kmeans 2 (n_g_ = 2)
Study 1	3	1
Study 2	3	1
Study 3	3	1
Study 4	1	2
Study 5	3	1
Study 6	2	2
Study 7	2	2
Study 8	3	1
Study 9	3	1
Study 10	3	1
Study 11	2	2
Study 12	3	1
Study 13	2	2
Study 14	3	1
Study 15	2	2
Study 16	3	1
Study 17	3	1
Study 18	3	1
Study 19	2	2
Study 20	1	2
Wc SSC	91.3%	71.8%
**IGDS9P**	**Kmeans 1** (n_g_ = 2)	
Study 1	1	-
Study 2	2	-
Study 3	1	-
Study 4	1	-
	83.1%	-
**IGDS27D**	**Kmeans 1** (n_g_ = 2)	
Study 1	2	-
Study 2	2	-
Study 3	2	-
Study 4	1	-
	93.8%	
**IGDS27P**	**Kmeans 1** (n_g_ = 2)	
Study 1	1	-
Study 2	2	-
Study 3	1	-
Study 4	1	-
	37.5%	-

**Table 4:** IGDS9D: dependence of cluster–covariable analysis.

	2 Clusters		3 Clusters	
	*c^2^* (df)	Cramer—V	*c^2^* (df)	Cramer—V
**Language**	16.38 ^NS^ (19)	0.373	4.97 ^NS^ (19)	0.343
**English language**	2.78 ^NS^ (19)	0.187	8.59 ^NS^ (19)	0.296
**Application mode**	5.87 ^NS^ (19)	0.308	9.28 ^NS^ (19)	0.341
**Sample**	7.69 ^NS^ (19)	0.334	6.26 ^NS^ (19)	0.268
**Gamer condition**	4.97 ^NS^ (19)	0.254	8.06 ^NS^ (19)	0.291

NS: not statistically significant (*p* > 0.50); Cramer-V: effect size estimate.

## Data Availability

Analysis script is available on request from the authors.

## Appendix A

The main difference between this study and Yoon et al. study [31] is that Yoon et al. focus only on the nine-item dichotomous response version of the IGDS. In our study, the four original versions [9] are analyzed, including the 9-item and 27-item versions with polytomous and dichotomous response.

The election of this instrument by Yoon et al. [31] is based on the results of the systematic review by King et al. [103], in which only the 9-item dichotomous and 27-item dichotomous and polytomous versions [103] were included (p. 4, Table 1). Therefore, not considering the four versions could mean an error in the search, since our sample (made in 2021 and updated in June 2022, consulting Scopus, WoS, PsycInfo, and Dialnet databases) already contains articles that use the not-included version (when the review was conducted in these and other databases by King et al. [103]).

All four versions of the IGDS report adequate reliability values in all four versions according to our systematic search. Although the nine-item dichotomous version is the most widely used in studies, some of them report inadequate reliability values, being the only version in which this occurs.

It should be noted that in our study, gray literature was included due to the small sample size. This fact can be both a strength, because it allows greater generalization of results by having a larger sample, and a weakness if it implies a reduction in quality. In the study by Yoon et al. [31], the quality criterion used was that the journal of publication had peer review, and therefore, we reviewed our gray literature and concluded that it also met this criterion. All the articles that formed part of the gray literature were published in peer-reviewed journals, thus providing a larger sample and more generalizable results.

Regarding the meta-analytic model used, Yoon et al. [31] used the random effects model while we used two models: the random coefficients model and the varying coefficients model. These two models were employed because the former is the generally accepted and preferred model in research, and the latter was appropriate because of the characteristics of the sample (the unlikely fulfillment of the assumption of normality of the hypothetical population of α coefficients, the actual absence of random selection of the manuscripts, and the small number of the selected studies).

Regarding heterogeneity assessment, in the study by Yoon et al. [31] Tau (τ), Tau-squared (τ^2^), and I^2^ were reported, whereas this study reported these measures plus the Q-statistic test and estimators of heterogeneity size R_b_ and CV_b_ and CV_w_.

Regarding the sources of heterogeneity analyzed, Yoon et al. [31] focus on sample type, study location, and instruments for measuring video game addiction. In our study, it is considered the language of the instrument, the mode of application, the type of sample, the total number of sample, and the presence of gamers/non-gamers in the sample. We also include a cluster analysis and a qualitative analysis of the significance of these clusters.

All of this highlights a greater rigor in conducting the meta-analysis and in assessing heterogeneity, as well as in exploring all versions of the IGDS. Thus, this study implies greater depth in the meta-analytic study of reliability with more focused generalizability.

## Appendix B

**Table A1:** Checklist for the corroboration of the meta-analytical report according to the REGEMA.

TITLE	Yes	No	NA
1. Title	X		
**ABSTRACT**			
2. Abstract	X		
**INTRODUCTION**			
3. Background	X		
4. Objectives	X		
**METHOD**			
5. Selection criteria	X		
6. Search strategies	X		
7. Data extraction	X		
8. Reported reliability	X		
9. Estimating the reliability induction and other sources of bias	X		
10. Data extraction of inducing studies	X		
11. Reliability of data extraction	X		
12. Transformation method	X		
13. Statistical model	X		
14. Weighting method	X		
15. Heterogeneity assessment	X		
16. Moderator analyses	X		
17. Additional analyses	X		
18. Software	X		
**RESULTS**			
19. Results of the study selection process	X		
20. Mean reliability and heterogeneity	X		
21. Moderator analyses	X		
22. Sensitivity analyses	X		
23. Comparison of inducing and reporting studies			X
24. Data set	X		
**DISCUSSION**			
25. Summary of results	X		
26. Limitations	X		
27. Implications for practice	X		
28. Implications for future research	X		
**FUNDING**			
29. Funding	X		
**PROTOCOL**			
30. Protocol	X		

NA: not applicable. Source: Adapted and based on the REGEMA checklist of Sanchez-Meca et al. [104].

## Appendix C

**Table A2:** Characteristics of the selected studies.

No.	Study	Version	Reported Reliability		Study Validity	Method	Reliability	Retest
			Available	Not Avail.				
1	Lemmens et al. (2015) [9]	IGDS9D	X		Empirical	α	0.83	
2	Sioni et al. (2017) [59]	IGDS9D	X		Induced	α	0.85	
3	Wartberg et al. (2017) [61]	IGDS9D	X		Induced	α	0.82	
4	Baiumy et al. (2018) [20]	IGDS9D	X		Induced	α	0.61	
5	Buiza-Aguado et al. (2018) [62]	IGDS9D	X		Induced	ω	0.81	
6	Koning et al. (2018) [71]	IGDS9D	X		Induced	α	0.74	
7	Van Den Eijnden et al. (2018) [73]	IGDS9D	X		Induced	α	0.73	
8	Brooks and Clark (2019) [63]	IGDS9D	X		Induced	α	0.82	
9	Dedeaux (2019) [69]	IGDS9D	X		Induced	α	0.84	
10	Stockdale et al. (2019) [60]	IGDS9D	X		Induced	α	0.81	
11	Grajewski et al. (2020) [64]	IGDS9D	X		Induced	α	0.69	
12	Lei et al. (2020) [18]	IGDS9D	X		Empirical	α	0.81	0.83
13	Wartberg et al. (2020) [24]	IGDS9D	X		Induced	α	0.66	
14	Zendle (2020) [74]	IGDS9D	X		Induced	α	0.79	
15	Booth et al. (2021) [68]	IGDS9D	X		Induced	α	0.74	
16	Liu et al. (2021) [65]	IGDS9D	X		Induced	α	0.83	
17	Oka et al. (2021) [15]	IGDS9D	X		Induced	α	0.84	
18	Paschke et al. (2021) [21]	IGDS9D	X		Empirical	α	0.78	
19	Paschke et al. (2021) [21]	IGDS9D	X		Empirical	α	0.72	
20	Paschke et al. (2021) [21]	IGDS9D	X		Empirical	α	0.56	
1	Lemmens et al. (2015) [9]	IGDS9P	X		Empirical	α	0.95	
2	Evren et al. (2017) [19]	IGDS9P	X		Empirical	α	0.93	0.756
3	Mills et al. (2018)	IGDS9P	X		Induced	α	0.85	
4	Lei et al. (2020) [18]	IGDS9P	X		Empirical	α	0.89	0.84
1	Lemmens et al. (2015) [9]	IGDS27D	X		Empirical	α	0.93	
2	Reyes et al. (2019) [72]	IGDS27D	X		Induced	α	0.9	
3	Ait Daoud (2020) [57]	IGDS27D	X		Induced	α	0.93	
4	Zemestani et al. (2021) [67]	IGDS27D	X		Induced	α	0.81	
1	Lemmens et al. (2015) [9]	IGDS27P	X		Empirical	α	0.94	
2	Evren et al. (2017) [19]	IGDS27P	X		Empirical	α	0.97	0.759
3	Allen and Anderson (2018) [22]	IGDS27P	X		Induced	α	0.96	
4	Gibbons and Bouldin (2019) [70]	IGDS27P	X		Induced	α	0.96	
5	Mills and Allen (2020) [66]	IGDS27P	X		Induced	α	0.96	

**Table A3:** Characteristics of the selected studies (frequency table).

No.	Study	V	Lang	English Language	Application Mode	N Sample	Sample	Gamer Condition
1	Lemmens et al. (2015) [9]	IGDS9D	Dutch	N	Self-report	989	Gen. Comm	Gamers
2	Sioni et al. (2017) [59]	IGDS9D	English	Y	Self-report	394	Gen. Comm	Gamers
3	Wartberg et al. (2017) [61]	IGDS9D	German	N	Interview	1020	Adolescents	Mixed
4	Baiumy et al. (2018) [20]	IGDS9D	Arabic	N	Self-report	204	Young	Gamers
5	Buiza-Aguado et al. (2018) [62]	IGDS9D	Spanish	N	Self-report	708	Adolescents	Mixed
6	Koning et al. (2018) [71]	IGDS9D	Dutch	N	Self-report	354	Adolescents	NR
7	Van Den Eijnden et al. (2018) [73]	IGDS9D	Dutch	N	Self-report	538	Adolescents	NR
8	Brooks and Clark (2019) [63]	IGDS9D	English	Y	Self-report	257	Gen. Comm	Mixed
9	Dedeaux (2019) [69]	IGDS9D	English	Y	Self-report	310	Gen. Comm	NR
10	Stockdale et al. (2019) [60]	IGDS9D	English	N	Encuesta	855	Adults	Mixed
11	Grajewski et al. (2020) [64]	IGDS9D	Polish	N	Self-report	1306	Gen. Comm	Gamers
12	Lei et al. (2020) [18]	IGDS9D	Chinese	N	Self-report	351	Gen. Comm	NR
13	Wartberg et al. (2020) [24]	IGDS9D	German	N	Interview	1001	Adolescents	Mixed
14	Zendle (2020) [74]	IGDS9D	English	Y	Self-report	1081	Gen. Comm	Mixed
15	Booth et al. (2021) [68]	IGDS9D	English	Y	Self-report	2078	Adults	Mixed
16	Liu et al. (2021) [65]	IGDS9D	Chinese	N	NR	1121	Adolescents	Mixed
17	Oka et al. (2021) [15]	IGDS9D	Japanese	N	Self-report	3938	Gen. Comm	Mixed
18	Paschke et al. (2021) [21]	IGDS9D	German	N	Interview	762	Adolescents	Gamers
19	Paschke et al. (2021) [21]	IGDS9D	German	N	Interview	777	Adolescents	Gamers
20	Paschke et al. (2021) [21]	IGDS9D	German	N	Interview	784	Adolescents	Gamers
1	Lemmens et al. (2015) [9]	IGDS9P	Dutch	N	Self-report	923	Gen. Comm	Gamers
2	Evren et al. (2017) [19]	IGDS9P	Turkish	N	Self-report	457	Young	Mixed
3	Mills et al. (2018)	IGDS9P	English	Y	Self-report	1029	Gen. Comm	Gamers
4	Lei et al. (2020) [18]	IGDS9P	Chinese	N	Self-report	378	Gen. Comm	NR
1	Lemmens et al. (2015) [9]	IGDS27D	Dutch	N	Self-report	989	Gen. Comm	Gamers
2	Reyes et al. (2019) [72]	IGDS27D	NR	NR	Self-report	1026	Gen. Comm	Gamers
3	Ait Daoud (2020) [57]	IGDS27D	English	Y	Self-report	423	Gen. Comm	Gamers
4	Zemestani et al. (2021) [67]	IGDS27D	Persian	N	Self-report	481	Gen. Comm	Mixed
1	Lemmens et al. (2015) [9]	IGDS27P	Dutch	N	Self-report	923	Gen. Comm	Gamers
2	Evren et al. (2017) [19]	IGDS27P	Turkish	N	Self-report	457	Young	Mixed
3	Allen and Anderson (2018) [22]	IGDS27P	English	Y	Self-report	315	Young	Gamers
4	Gibbons and Bouldin (2019) [70]	IGDS27P	English	Y	Self-report	272	Young	NR
5	Mills and Allen (2020) [66]	IGDS27P	English	Y	Self-report	487	Gen. Comm	Gamers

N: no; Y: yes; NR: not reported; V: version; Lang: language; Gen. Comm.: general community.
